# Maintaining the Mitochondrial Quality Control System Was a Key Event of Tanshinone IIA against Deoxynivalenol-Induced Intestinal Toxicity

**DOI:** 10.3390/antiox13010121

**Published:** 2024-01-18

**Authors:** Cong Zhang, Youshuang Wang, Xinyu Zhang, Kefei Zhang, Fengjuan Chen, Jiayan Fan, Xuebing Wang, Xu Yang

**Affiliations:** 1 College of Veterinary Medicine, Henan Agricultural University, Zhengzhou 450002, China; zhangcong@henau.edu.cn (C.Z.); yswang@stu.henau.edu.cn (Y.W.); 19545646110@163.com (X.Z.); 15139070731@163.com (K.Z.); fengjuanchen@stu.henau.edu.cn (F.C.); fanjiayan2022@163.com (J.F.); xbwang74@163.com (X.W.); 2 Key Laboratory of Quality and Safety Control of Poultry Products, Ministry of Agriculture and Rural Affairs, Zhengzhou 450002, China

**Keywords:** Tanshinone IIA, deoxynivalenol, mitochondrial quality control, IPEC-J2 cells

## Abstract

Deoxynivalenol (DON) is the one of the most common mycotoxins, widely detected in various original foods and processed foods. Tanshinone IIA (Tan IIA) is a fat-soluble diterpene quinone extracted from *Salvia miltiorrhiza* Bunge, which has multi-biological functions and pharmacological effects. However, whether Tan IIA has a protective effect against DON-induced intestinal toxicity is unknown. In this study, the results showed Tan IIA treatment could attenuate DON-induced IPEC-J2 cell death. DON increased oxidation product accumulation, decreased antioxidant ability and disrupted barrier function, while Tan IIA reversed DON-induced barrier function impairment and oxidative stress. Furthermore, Tan IIA dramatically improved mitochondrial function via mitochondrial quality control. Tan IIA could upregulate mitochondrial biogenesis and mitochondrial fusion as well as downregulate mitochondrial fission and mitochondrial unfolded protein response. In addition, Tan IIA significantly attenuated mitophagy caused by DON. Collectively, Tan IIA presented a potential protective effect against DON toxicity and the underlying mechanisms were involved in mitochondrial quality control–mediated mitophagy.

## 1. Introduction

Mycotoxin contamination has already become a global public health issue, especially in developing countries. Globally, one in four cereals are contaminated with mycotoxins, as estimated by the Food and Agriculture Organization of the United Nations [1]. One of the most typical mycotoxins is deoxynivalenol (DON), which is commonly found in processed foods, animal-derived products, corn, barley, wheat and nuts [2]. Epidemiological reports in Europe (France, Belgium, Norway and Britain) have indicated that over 80% of urine samples from adults and children contain DON or its metabolites, and the average concentration ranges from 7.0 to 120.0 μg/L [3,4]. It does not persistently threaten human and livestock health but causes enormous economic losses. Once ingested, digestive system dysfunction is the main cause of DON poisoning, mainly manifested as nausea, vomiting and anorexia, weight loss and even death in humans and animals [5]. Thus, the intestine serves as the primary organ for DON interaction, processing, and ingestion. It also serves as the initial barrier against DON attack. Intestinal impairments are typically linked to food rejection, nausea, and vomiting. There is a consensus among researchers that DON can damage intestinal structure and intestinal barrier function, disturb intestinal microbial composition and cause systemic toxicity [6,7]. Hence, maintaining the function of intestinal epithelial cells and reducing the absorption and toxic effect of DON are strategically important for controlling/mitigating the toxic effect of DON.

Mitochondria are the “powerhouses” of cells because they act as metabolic hubs and signaling platforms for energy supply [8,9]. It is also well known that mitochondria are involved in various types of programmed/nonprogrammed cell death (such as apoptosis, necrosis, ferroptosis, etc.) as the central executioners [8]. Therefore, mitochondrial quality must be well controlled to maintain cellular homeostasis. Under diverse stresses, mitochondria may face an inherent challenge that increases their likelihood of dysfunction. It is imperative to utilise multiple quality control strategies to identify, monitor, repair, or eliminate dysfunctional mitochondria in response to mitochondrial fission and fusion, intercellular mitochondrial transfer, mitochondrial biogenesis, mitochondrial unfolded protein response (UPRmt) and mitophagy [10,11]. Emerging evidence has linked mitochondrial quality control (MQC) disorder with several distinct diseases (including gastrointestinal diseases, cardiovascular diseases, diabetes, neurological diseases, etc.) [12,13,14,15]. Intestinal tissue is tissue with high turnover, and intestinal epithelial cells are entirely regenerated every 3 to 5 days to protect against mucosal damage and infection [16]. In this process, intestinal epithelial cells need to be rapidly differentiated from intestinal stem cells, and these phenotypic changes are dependent on mitochondrial function [16]. Thus, mitochondria are abundant and play a vital role in intestinal epithelial cells. Recent studies have suggested that mitochondria act as energy metabolism regulators in intestinal epithelial cells, which obtain energetic sources to form an enormous evolutionary advantage via mitochondrial oxidative phosphorylation [17]. Healthy mitochondria can regulate ion transport, inhibit oxygenation of colonic epithelial cells and sustain an oxygen environment for the intestinal cavity [18,19]. In addition, the decreased mitochondrial viability of epithelial cells will upregulate Nos2 gene expression, which promotes the intestinal inflammatory response [18,20]. In our previous study, DON treatment caused mitochondrial dysfunction by opening the mitochondrial permeability transition pore and eliminating mitochondrial membrane potential, causing mitochondrial fission/fusion imbalance and inducing mitophagy in IPEC-J2 cells. In view of the role of mitochondria in maintaining intestinal energy metabolism, iron-sulfur cluster biosynthesis, calcium buffering, redox balance, inflammatory response, cell survival of intestinal epithelial cells and the enterotoxic effect of DON [9], there is reason to believe that mitochondrial dysfunction may be the dominant event in DON-mediated intestinal toxicity [1]. Therefore, alleviating mitochondrial dysfunction and maintaining mitochondrial homeostasis may be potential targeted therapeutic strategies for antagonising DON enterotoxicity.

*Salvia miltiorrhiza* Bunge’s most prevalent active ingredient is Tanshinone IIA (Tan IIA), a fat-soluble quinone diterpene. Tan IIA has antioxidant, anti-inflammatory, antifibrotic, antiviral, antitumour and antiplatelet aggregation effects. Tan IIA possesses a characteristic quinone-type structure, facilitating its participation in redox reactions and electron transfer [21]. In Asia, commercialized Tan IIA and its derivatives have been widely applied to the clinical treatment of cerebrovascular diseases and cardiovascular diseases. Meanwhile, additional pharmacological effects of Tan IIA are being discovered and explored. It has been successively reported that Tan IIA has a variety of pharmacological activities on the urinary, anticarcinogenic, nervous, respiratory, digestive and motor systems [22]. Tan IIA protected the small intestine of septic rats by inhibiting apoptosis of intestinal epithelial cells and reducing the activation of inflammatory cytokines [23]. Tan IIA alleviated the occurrence of colon tumors by suppressing intestinal inflammation [21]. The inhibition of neutrophil migration and activation by Tan IIA mitigated inflammation-induced colitis in mice [24]. Tan IIA treatment for human colon cancer simultaneously promoted the growth of beneficial symbiotic bacteria in the intestinal flora, especially Bifidobacterium longum [25]. Tan IIA’s protective effect against LPS-induced small intestine injury may be attributed to its ability to inhibit inflammatory factors and enhance autophagy [26]. In our previous studies, Tan IIA was found to improve intestinal epithelial cell damage induced by DON by modulating the cell pyroptosis signaling pathway [27]. During the research process, we observed a significant impact of Tan IIA on the mitochondrial-related genes of IPEC-J2 cells. Therefore, we shifted our focus to mitochondria to investigate whether Tan IIA mitigates DON-induced intestinal injury by regulating mitochondrial-related genes.

The purpose of this study was to explore the protective effect and antagonistic mechanism of Tan IIA on intestinal epithelial cell injury induced by DON. This research could provide a new detoxification strategy for DON-induced intestinal toxicity and broaden the pharmacological application of the traditional Chinese medicinal plant *Salvia miltiorrhiza* Bunge.

## 2. Materials and Methods

### 2.1. Cell Culture and Drug Intervention

IPEC-J2 cells were obtained from Tongpai Biotechnology Co., Ltd. Shanghai, China, and were maintained in DMEM (Gibco, Billings, MT, USA) supplemented with 1% penicillin-streptomycin and 10% FBS (CellMax, Beijing, China). When IPEC-J2 cells as a monolayer reached 70–80% confluence, the cells were incubated with 0, 15, 30, 45, 60, or 75 μg/mL Tan IIA or/and 1 μM DON (Pribolab, Qingdao, China). Tan IIA (HPLC ≥ 98%) was purchased from Victory Biological Technology Co., Ltd. (Sichuan, China). After 24 h of incubation, the cells and supernatants were collected for subsequent experiments. 

### 2.2. Cytotoxicity Measurement

Cell viability was measured by a CCK8 assay kit (APExBIO, Houston, TX, USA). IPEC-J2 cells were seeded at a density of 2 × 10^5^ cells/well in a 96-well plate. First, the cells were incubated with different concentrations of Tan IIA (0, 15, 30, 45, 60 and 75 μg/mL) to select the optimal concentration of Tan IIA. Then, the cells were incubated with 1 μM DON and/or optimal Tan IIA to explore the protective effect of Tan IIA on DON-induced IPEC-J2 cell damage. Finally, the cell viability value was analysed by a microplate reader (TECAN, Männedorf, Switzerland).

Lactate dehydrogenase (LDH) release was measured by an LDH cytotoxicity kit (Beyotime, Shanghai, China). After incubation with 1 μM DON and/or 45 μg/mL Tan IIA, the supernatants were collected and treated with LDH reagents. Finally, LDH release was analysed by a microplate reader (TECAN, Switzerland).

### 2.3. Cell Redox State Measurement

The cell redox state was measured by an ROS detection kit (Beyotime, China), MDA (Solarbio, Beijing, China), T-AOC, SOD and CAT (Jiancheng, Nanjing, China). According to the instructions, the cell lysis solution and living cells were prepared as described in a previous study [28]. The fluorescence of DCFH-DA and enzyme activity were analysed using a fluorescence microscope and microplate reader, respectively.

### 2.4. MPTP Opening and MMP Measurement

MPTP was visualized by fluorescence microscopy after staining with a combination of calcein AM, CoCl_2_ and/or quenching reagents (Beyotime Biological Technology, Shanghai, China). The cell staining method was the same as previously described. Green calcein AM fluorescence was observed at a 505 nm emission wavelength. The opening of the MPTP in IPEC-J2 cells was judged by the change in fluorescence intensity, and strong green fluorescence indicated that the MPTP was normal.

MMP was visualized by fluorescence microscopy after staining with a JC-1 test kit (Beyotime Biological Technology, Shanghai, China). The cell staining method was the same as previously described [28]. Red aggregated JC-1 was observed at a 580 nm emission wavelength, and green monomeric JC-1 was observed at a 525 nm emission wavelength. The levels of MMP in IPEC-J2 cells were judged by fluorescence intensity; strong red fluorescence indicated that the MMP was normal, and strong green fluorescence indicated that the MMP was destroyed.

### 2.5. RNA Extraction and Quantitative RT–PCR Analysis

RNA was isolated from IPEC-J2 cells using TRIzol reagent and prepared for cDNA synthesis. qRT–PCR was performed on a qTOWER^®^ (Analytik Jena, Jena, Germany) system using SYBR Mix (Vazyme, Nanjing, China) and calculated by the 2^−ΔΔCt^ method. The primer sequences are shown in Table S1.

### 2.6. Western Blot Analysis

Protein isolated from IPEC-J2 cells was separated by SDS gel electrophoresis and transferred to PVDF membranes. Then, membranes were incubated with tight junction primary antibodies (Claudin-1, Claudin-3, ZO-1, N-cad and Occludin) (Biosynthesis Biotechnology Inc., Beijing, China), mitophagy primary antibodies (LC3, P62, Beclin1, Parkin and PINK1) (ABclonal, Wuhan, China), a housekeeping antibody (β-actin) and a secondary antibody (ABclonal, Wuhan, China), respectively. Finally, the bands were quantified using a Tanon-5200 (Tanon, Shanghai, China) [29,30].

### 2.7. Statistical Analysis

Statistical analysis was conducted by a one-way analysis of variance using GraphPad Prism 8.0.1 software. PCA was conducted using SPSS 26 software. The data are presented as the mean ± SEM. *p* < 0.05 was considered to be statistically significant.

## 3. Results

### 3.1. Tan IIA Alleviates the Cytotoxicity Caused by DON in IPEC-J2 Cells

**Figure 1:**
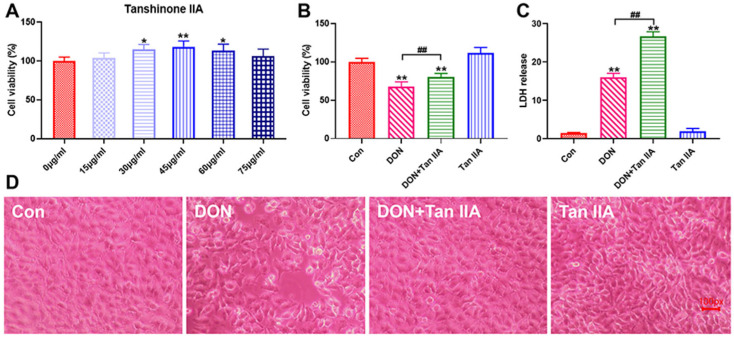
Effect of DON or/and Tan IIA exposure on cell viability, LDH release and morphology of IPEC-J2 cells. (**A**) Cell viability of IPEC-J2 treated with Tan IIA (0, 15, 30, 45, 60 and 75 μg/mL) for 24 h. (**B**) Cell viability of IPEC-J2 treated with DON or/and Tan IIA for 24 h. (**C**) LDH release of IPEC-J2 cells. (**D**) Morphological observations of IPEC-J2 (red bar = 100 px). All data represent the mean ± SEM. * *p* < 0.05; ** *p* < 0.01 vs. Con group; ## *p* < 0.01 vs. DON group.

The cell activity test was executed to determine the safe concentration of Tan IIA for IPEC-J2 cells. The CCK8 results showed that 45 µg/mL Tan IIA had the strongest proliferative activity on IPEC-J2 cells (Figure 1A) and significantly alleviated the cell activity decrease caused by DON (*p* < 0.01) (Figure 1B). The LDH release test further confirmed that Tan IIA can relieve the cytotoxicity of DON (*p* < 0.01) (Figure 1C). Meanwhile, discrete cell arrangement, decreased cell density and enlarged gaps were observed in the DON treatment group. Tan IIA significantly relieved the cell morphological damage caused by DON, and less damage to cell integrity was observed in the DON + Tan IIA group (Figure 1D). Taken together, Tan IIA can significantly alleviate the cytotoxicity induced by DON.

### 3.2. Tan IIA Alleviates the Barrier Function Impairment Caused by DON in IPEC-J2 Cells

**Figure 2:**
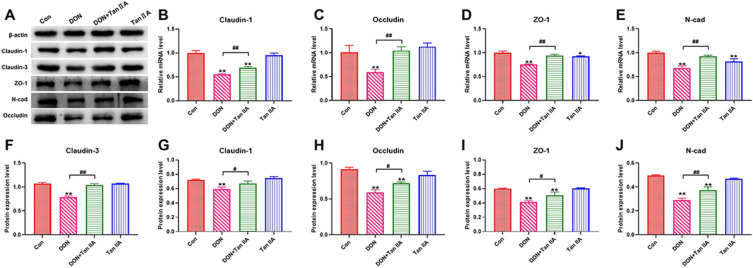
Effects of DON or/and Tan IIA exposure on barrier function in IPEC-J2 cells. (**A**) Western blotting bands of tight junction proteins. (**B**) Relative mRNA levels of Claudin-1. (**C**) Relative mRNA levels of Occludin. (**D**) Relative mRNA levels of ZO-1. (**E**) Relative mRNA levels of N-cad. (**F**) Relative protein levels of Claudin-3. (**G**) Relative protein levels of Claudin-1. (**H**) Relative protein levels of Occludin. (**I**) Relative protein levels of ZO-1. (**J**) Relative protein levels of N-cad. All data represent the mean ± SEM. * *p* < 0.05; ** *p* < 0.01 vs. Con group; # *p* < 0.05; ## *p* < 0.01 vs. DON group.

The protein expression of Claudin-1, Occludin, ZO-1 and N-cad was significantly inhibited in the DON group compared with the Con group (*p* < 0.01) (Figure 2A,F–J). Tan IIA treatment significantly attenuated the inhibition of Claudin-1, Occludin, ZO-1 and N-cad protein expression (*p* < 0.01). The mRNA expression of Claudin-3, Claudin-1, Occludin, ZO-1 and N-cad was significantly decreased in the DON group compared with the Con group (*p* < 0.01) (Figure 2B–E) but significantly boosted in the DON + Tan IIA group compared with the DON group (*p* < 0.05). Taken together, these results demonstrate that Tan IIA mitigated the impairment of IPEC-J2 cell barrier function induced by DON.

### 3.3. Tan IIA Alleviates the Oxidative Damage Caused by DON in IPEC-J2 Cells

**Figure 3:**
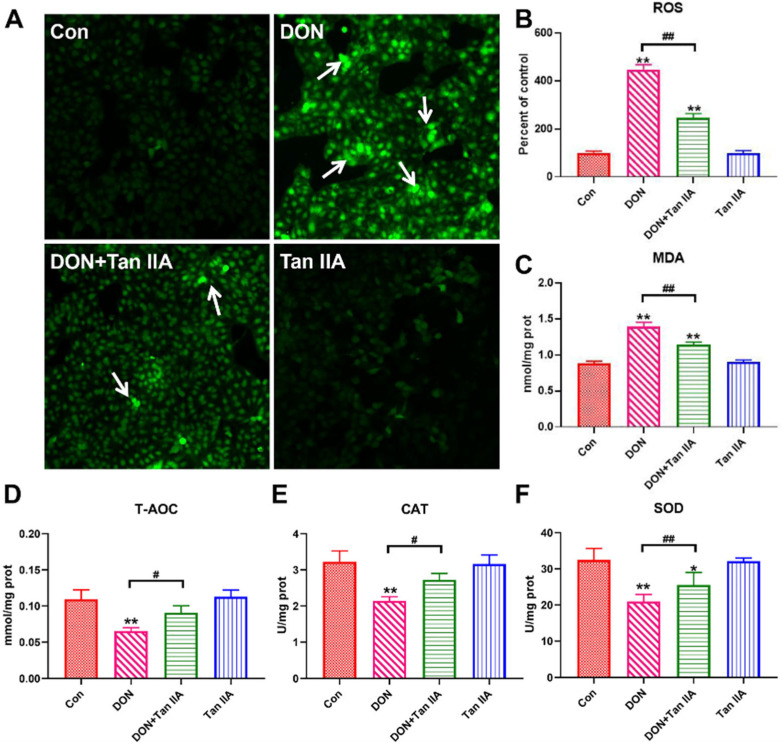
Effects of DON or/and Tan IIA exposure on redox state in IPEC-J2 cells. (**A**) ROS fluorescence. White arrows indicate ROS-positive cells. Magnification: 100×. (**B**) Quantitative analysis of ROS fluorescence intensity. (**C**) MDA content. (**D**) T-AOC level. (**E**) CAT activity. (**F**) SOD activity. All data represent the mean ± SEM. * *p* < 0.05; ** *p* < 0.01 vs. Con group; # *p* < 0.05; ## *p* < 0.01 vs. DON group.

To evaluate the antioxidant effect of Tan IIA on DON-induced IPEC-J2 cell oxidative damage, we measured intracellular ROS production and lipid peroxidation levels using a DCFH-DA probe and MDA kit. As shown in Figure 3A–C, ROS fluorescence intensity and MDA content were significantly elevated in the DON group compared with the Con group (*p* < 0.01). However, ROS fluorescence intensity and MDA content were significantly suppressed in the DON + Tan IIA group compared with the DON group. In contrast, the level of T-AOC as well as the activity of SOD and CAT were markedly decreased in the DON group compared with the Con group (*p* < 0.01) (Figure 3D–F), but these alterations were partially or completely restored by Tan IIA supplementation. Taken together, these results demonstrated that Tan IIA alleviated DON-induced IPEC-J2 cell oxidative damage.

### 3.4. Tan IIA Alleviates the Mitochondrial Dysfunction Caused by DON in IPEC-J2 Cells

**Figure 4:**
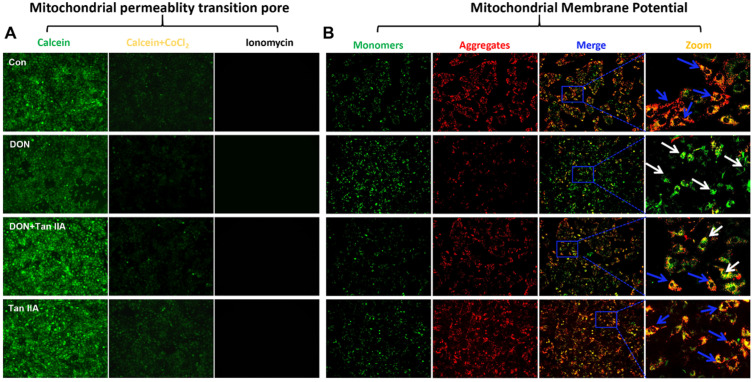
Effects of DON or/and Tan IIA exposure on mitochondrial function in IPEC-J2 cells. (**A**) Measurement of MPTP. (**B**) Measurement of MMP. The blue arrows indicate JC-1 aggregates; the white arrows indicate JC-1 monomers. Magnification: 100×.

Mitochondrial dysfunction has been considered a fundamental mechanism during cell damage. To investigate the protective mechanism of Tan IIA against DON-induced damage to IPEC-J2 cells, we examined the opening of MPTP and alterations in MMP in IPEC-J2 cells. As shown in Figure 4A,B, the fluorescence of calcein and JC-1 aggregates declined, while the fluorescence of JC-1 monomers were enhanced in IPEC-J2 cells after treatment with DON. Compared with the DON group, the fluorescence of calcein and JC-1 aggregates in the DON + Tan IIA group were higher, but the fluorescence of JC-1 monomers was lower, suggesting that Tan IIA can inhibit the opening of MPTP and the decrease of MMP. Taken together, these results demonstrated that Tan ⅡA alleviated DON-induced IPEC-J2 cell mitochondrial dysfunction.

### 3.5. Tan IIA Alleviates the MQC Disorder Caused by DON in IPEC-J2 Cells

**Figure 5:**
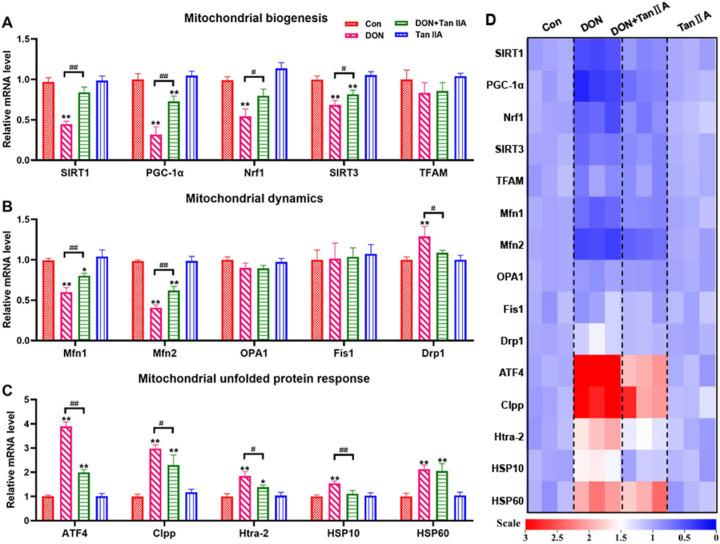
Effects of DON or/and Tan IIA exposure on mitochondrial quality control in IPEC-J2 cells. (**A**) Relative mRNA levels of mitochondrial biogenesis–related factors (SIRT1, PGC-1α, Nrf1, SIRT3and TFAM). (**B**) Relative mRNA levels of mitochondrial dynamics–related factors (Mfn1, Mfn2, OPA1, Drp1 and Fis1). (**C**) Relative mRNA levels of mitochondrial unfolded protein response–related factors (ATF4, Clpp, Htra-2, HSP10 and HSP60). (**D**) Heat map of relative mRNA levels of mitochondrial quality control–related genes. All data represent the mean ± SEM. * *p* < 0.05; ** *p* < 0.01 vs. Con group; # *p* < 0.05; ## *p* < 0.01 vs. DON group.

MQC is the self-defense mechanism of mitochondria to ensure their relative stability. To investigate the protective mechanism of Tan IIA on DON-induced mitochondrial dysfunction in IPEC-J2 cells, the mRNA levels of genes associated with mitochondrial biogenesis, mitochondrial dynamics and mitochondrial unfolded protein response–related genes were detected. As shown in Figure 5A–D, DON and Tan ⅡA had no effects on the mRNA levels of TFAM, OPA1, or Fis1 (*p* > 0.05). However, the mRNA levels of SIRT1, PGC-1α, Nrf1, SIRT3, Drp1, ATF4, Clpp, Htra-2 and HSP10 were shown to be lower in the DON group than in the Con group (*p* < 0.01), and Tan ⅡA partially or completely restored these changes back to normal in the DON + Tan ⅡA group (*p* < 0.05). In addition, the mRNA levels of Mfn1 and Mfn2 were higher in the DON group than in the Con group (*p* < 0.01), while cotreatment with Tan ⅡA significantly alleviated the decreases in these genes (*p* < 0.01). Taken together, these results demonstrated that Tan ⅡA alleviated DON-induced IPEC-J2 cell MQC disorder.

### 3.6. Tan IIA Alleviates IPEC-J2 Cell Mitophagy Caused by DON

**Figure 6:**
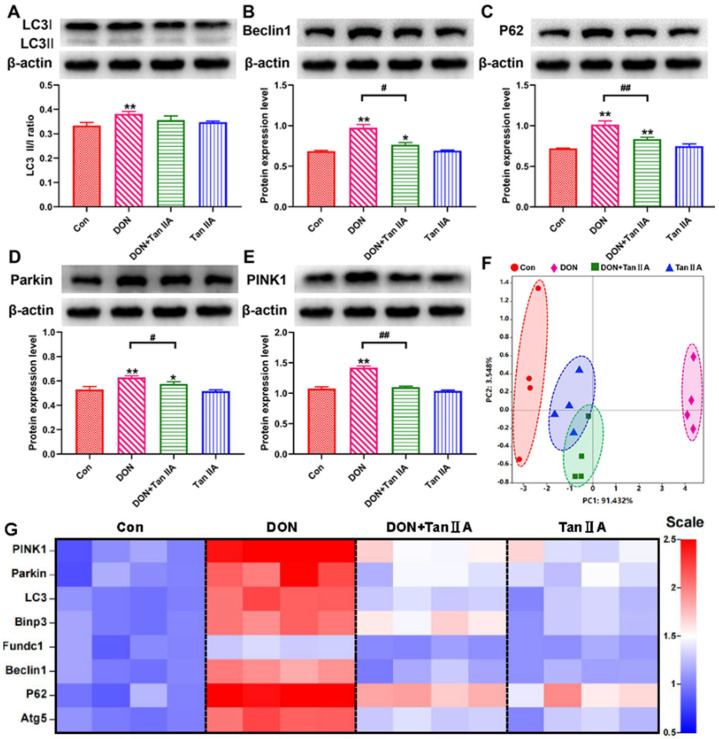
Effects of DON or/and Tan IIA exposure on mitophagy in IPEC-J2 cells. (**A**) Relative protein level of LC3. (**B**) Relative protein level of Beclin1. (**C**) Relative protein level of P62. (**D**) Relative protein level of Parkin. (**E**) Relative protein level of PINK1. (**F**) PCA score plot results comparing the mitophagy–related gene levels of four treatment groups. (**G**) Heat map of relative mRNA levels of mitophagy–related genes. All data represent the mean ± SEM. * *p* < 0.05; ** *p* < 0.01 vs. Con group; # *p* < 0.05; ## *p* < 0.01 vs. DON group.

To get additional insight into the protective mechanism of Tan ⅡA in preventing DEHP exposure-induced MQC disorder, the expression of mitophagy-related proteins and genes was investigated. The protein expression levels of PINK1, Parkin, LC3, p62 and Beclin-1 in IPEC-J2 cells were higher in the DON group than in the Con group (*p* < 0.01) (Figure 6A–E). However, Tan IIA treatment significantly decreased the expression of PINK1, Parkin, LC3, p62 and Beclin-1 compared with the DON group (*p* < 0.05; *p* < 0.01). Similarly, the mRNA levels of Bnip3, Fundc1, LC3, P62, Atg5, PINK1, Parkin and Beclin1 showed the same trend (Figure 6G). Finally, PCA of mitophagy-related genes was used to reveal the differences among the Con, DON, Tan ⅡA and DON + Tan ⅡA groups. A longer distance was presented between the Con group and the DON group, and a shorter distance was presented between the Tan ⅡA or DON + Tan ⅡA group and the Con group (PC1 = 91.432%, PC2 = 3.548%) (Figure 6F and Table S2). Taken together, these results exhibited that Tan ⅡA mitigated DON-induced mitophagy in IPEC-J2 cells.

## 4. Discussion

DON is a common feed toxin that is mainly absorbed into the body through the digestive tract. The intestine becomes the first barrier against DON entry into the body and the main target of DON toxicity. Consequently, it is especially crucial to investigate certain natural substances that have been converted from conventional and ethnic medicine in order to preserve intestinal health, lessen DON’s toxicity to the digestive tract, or prevent DON’s negative effects. Tan IIA is the main extract and active ingredient of *Salvia miltiorrhiza* Bunge, which is mainly distributed in China and its neighbouring countries. In Asia, *Salvia miltiorrhiza* Bunge is considered a classic Chinese herb for treating cardiovascular disorders, stroke, diabetes and Alzheimer’s disease, which are closely related to mitochondrial quality [31,32,33]. Recent studies have found that the concentration of Tanshinone in the digestive tract is higher than that in the cardiovascular system after oral administration, suggesting that Tan IIA may play a role in intestinal health [34]. Our previous research has indicated that Tan IIA mitigates cell pyroptosis in DON-induced IPEC-J2 cells by inhibiting the accumulation of ROS and the activation of NLRP3 inflammasomes [27]. Since damaged mitochondria are the primary sites for ROS generation, excessive ROS accumulation can trigger oxidative stress and activate NLRP3 inflammasomes [35,36,37]. Therefore, we speculate that the mechanism by which Tan IIA mitigates the toxicity of DON-induced IPEC-J2 cells is likely achieved through its impact on mitochondrial function. In this study, Tan IIA alleviated intestinal damage induced by DON by maintaining MQC (mitochondrial biogenesis, UPRmt, mitochondrial dynamics, PINK1-mediated mitophagy) in IPEC-J2 cells and was expected to be a candidate drug for alleviating DON toxicity.

The CCK-8 assay verified that Tan IIA could stimulate the proliferation of IPEC-J2 cells when the concentration of Tan IIA was present in concentrations less than 60 μg/mL. Tan IIA (40 μg/mL) had the best proliferative activity and was selected for subsequent testing. Subsequently, it was proven that Tan IIA could effectively relieve the DON-induced decrease in cell viability and LDH release, proving that Tan IIA can alleviate DON-induced cytotoxicity. Abnormal cell morphology and increased space often indicate damaged cell structure and intercellular connections. In this study, DON treatment increased the intercellular space and caused cells to have a rounded or irregular shape. Our previous study found that DON exposure can suppress the expression of Claudin-1, ZO-1, N-cad and Occludin, suggesting that DON disrupted the connections between IPEC-J2 cells. However, further detection of tight junction proteins revealed that Tan IIA ameliorates the suppressive effect on Claudin (Claudin-1 and Claudin-3), ZO-1, N-Cad and Occludin expression induced by DON in the current study. Additional evidence suggests that Tan IIA can alleviate DON’s cytotoxicity on IPEC-J2 cells. Based on these findings, it was established that Tan IIA protected IPEC-J2 cells against DON-induced cell death and intestinal barrier disruption.

It has been widely demonstrated that trichothecene can alter the integrity of the membrane by disrupting the cellular antioxidant system and inducing lipid peroxidation or ROS accumulation [38]. ROS are made up of superoxide anions, hydroxyl radicals and their byproducts, which can induce protein oxidation, DNA fragmentation and lipid peroxidation. MDA is the product of cell lipid oxidation that reflects the identifying severity of cell membrane damage. SOD and CAT are considered the most important enzymes in eliminating ROS. The former can catalyse superoxide O_2_^−^ into H_2_O_2_. Subsequently, the latter decomposes H_2_O_2_ into H_2_O and oxygen [39,40,41]. A previous study found that different concentrations of DON could result in ROS production and decreases in CAT activity, apoptosis and inflammation in IPEC-J2 cells. The excess ROS and apoptosis induced by DON could be eliminated by NAC (an antioxidant), suggesting that DON caused IPEC-J2 cell damage by promoting excessive ROS production [38]. Similarly, ROS and MDA levels were markedly increased, but the levels of the antioxidant markers T-AOC, CAT and SOD were significantly decreased in IPEC-J2 cells exposed to DON in the current study. Moreover, we found that Tan IIA supplementation could significantly inhibit ROS and MDA accumulation and relieve the levels of T-AOC, SOD and CAT. These findings indicate that Tan IIA treatment mitigated DON-induced oxidative stress by eliminating oxidation product accumulation and enhancing antioxidative ability in IPEC-J2 cells.

**Figure 7:**
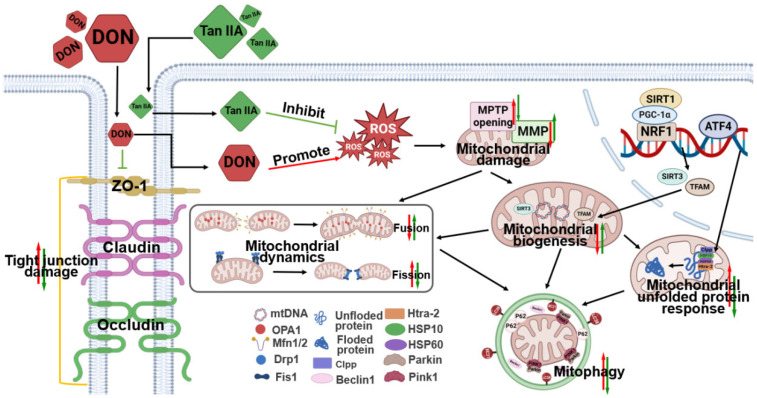
The mechanism diagram illustrating the alleviation of deoxynivalenol-induced intestinal toxicity by Tanshinone IIA through its stabilisation of the mitochondrial quality control system. DON disrupts tight junctions and generates excessive ROS in IPEC-J2 cells. Excessive ROS can damage mitochondria, including reduction of MMP and increased opening of MPTP. DON leads to a decrease in mitochondrial biogenesis and disrupts mitochondrial dynamics, including increased mitochondrial fusion and decreased mitochondrial fission, as well as an increase in mitochondrial unfolded protein response. However, Tan IIA can reverse various damages caused by DON.

One of the underlying pathogenic processes in intestinal diseases is associated with the deterioration of mitochondrial dysfunction and related oxidative stress [1,42,43]. Mitochondria are not only key organelles for maintaining intestinal epithelial homeostasis but also toxic targets of DON exposure [1]. A recent study demonstrated that DON exposure significantly increased the percentage of fragmented mitochondria and decreased the MMP, as visualized by MitoTracker Red probe and TMRE staining [44]. This is also the case in this study; DON exposure resulted in a reduction in MMP accompanied by continuous opening of the MPTP. Intriguingly, our data showed that Tan IIA effectively protected against DON-induced mitochondrial impairment. To explore the potential of Tan IIA to rescue DON-induced mitochondrial impairment, we next investigated the effect of Tan IIA and/or DON on the MQC system. The MQC system is an advantageous evolution for mitochondria to maintain a complex and healthy network to meet cellular requirements, cope with various stresses and respond to physiological adaptations [14]. When mitochondria are challenged, they will initiate multiple MQC mechanisms to recover their homeostasis. First, the UPRmt, a retrograde molecular signal to the nucleus, is activated to degrade misfolded proteins by transcription of mitochondrial chaperones and proteases. Then, mitochondria change their own morphology by mitochondrial dynamics to repair damaged mitochondrial components. Mitochondrial fusion promotes communication and cooperation between healthy and unhealthy mitochondria by component exchange, while mitochondrial fission promotes mitochondrial fragment formation by separating damaged mitochondria. Finally, irreparable mitochondria are engulfed and degraded by lysosomes through mitophagy. Meanwhile, mitochondrial materials are renewed via mitochondrial biogenesis to generate new mitochondrial offspring [11,45]. However, if the challenge exceeds the mitochondrial tolerance or the MQC system is destroyed, mitochondria will trigger danger signals, resulting in mitochondrial dysfunction and even cell death. It has been demonstrated that DON induces mitochondrial dysfunction by disrupting mitochondrial dynamics and mitochondrial biogenesis in various cells and tissues. Ji et al. found that DON decreased PGC-1α, TFAM and Nrf1 mRNA expression, causing very pronounced hepatoxicity in piglets [46]. Ma et al. found that DON increased MFF mRNA expression but decreased Mfn2 and OPA1 mRNA expression, causing mitochondrial morphology alterations in the kidney [47]. Our previous study showed that DON exposure decreased the protein and mRNA expression of mitochondrial fusion factors (Mfn1, Mfn2 and OPA1) and increased mitochondrial fission factors (Drp1, Fis1, MIEF1 and MFF) in IPEC-J2 cells. However, no study has explored the effect of DON on the UPRmt process. In the current study, DON inhibited mitochondrial biogenesis, disrupted mitochondrial dynamics balance and triggered the UPRmt process in IPEC-J2 cells. Tan IIA could completely or partially restore mitochondrial function alterations induced by DON. Mitophagy is another key step in the MQC system. It can specifically remove damaged mitochondria to reduce cell harm. Studies have shown that DON significantly increases PINK1, Bnip3, P62 and LC3 mRNA levels in the kidney and ileum [47,48]. In our previous study, IPEC-J2 cells could initiate PINK1/Parkin-mediated mitophagy under DON stress. In the current research, the upregulated protein expression of PINK1, Parkin, p62, Beclin-1 and LC3 and the mRNA expression of Bnip3, Fundc1, LC3, P62, Atg5, PINK1, Parkin and Beclin1 were ameliorated by Tan IIA treatment, indicating that Tan IIA reduces the mitophagy level triggered by DON. Based on the above evidence, Tan IIA has a positive effect on the UPRmt process, mitochondrial biogenesis, mitochondrial dynamics and mitophagy, indicating that it could ameliorate DON-induced mitochondrial impairment by regulating the MQC system in IPEC-J2 cells (Figure 7).

## 5. Conclusions

In conclusion, DON causes intestinal epithelial cell injury, including cell morphology arrangement, oxidative stress, barrier dysfunction and even cell death. However, Tan IIA can ameliorate the IPEC-J2 cell toxicity induced by DON, which is attributed to the positive effect of Tan IIA on regulation of the MQC system. Tan IIA is able to maintain MMP, ensure the balance of mitochondrial fission/fusion and regulate the process of mitochondrial biogenesis, UPRmt and Pink1-mediated mitophagy. This study provides new evidence about the potential mechanism underlying the effect of Tan IIA on DON-induced IPEC-J2 cell toxicity and broadens the medicinal application potential of Tanshinone.

## Data Availability

Data will be made available on request.

## Supplementary Materials

The following supporting information can be downloaded at: https://www.mdpi.com/article/10.3390/antiox13010121/s1. Table S1. Sequences of oligonucleotide primers for qRT-PCR. Table S2. Total Variance Explained of PCA. Figure S1. Original western blot gels used in the study.
