# Supplementary material for: Maintaining the Mitochondrial Quality Control System Was a Key Event of Tanshinone IIA against Deoxynivalenol-Induced Intestinal Toxicity
Source: Antioxidants (Basel). 2024 Jan 18;13(1):121. doi: 10.3390/antiox13010121 (PMC10812604; doi:10.3390/antiox13010121)

**Table S1. Sequences of oligonucleotide primers for qRT-PCR.**

| Gene Names | Sequence (5' → 3') | NCBI Reference Sequence | Amplicon size (bp) |
|---|---|---|---|
| GAPDH1 | GTCGGAGTGAACGGATTTGGC<br>CTTGCCGTGGGTGGAATCAT | NM_001289745.3 | 150 |
| Occludin | GACAGACTACACAACTGGCGG<br>TGTACTCCTGCAGGCCACTG | NM_001302926.2 | 662 |
| N-Cad | CTTCATGCTGGTGGTGTCCTTGG<br>CACAGTCTTTGGCAGGGCTCAG | NM_000165.5 | 149 |
| Claudin1 | CCATCGTCAGCACCGCACTG<br>CGACACGCAGGACATCCACAG | NM_001244539.1 | 107 |
| ZO-1 | CCTGAGTTTGATAGTGGCGTTGA<br>AAATAGATTTCCTGCCCAATTCC | XM_003353439.2 | 269 |
| SIRT1 | ACTCTCCCTCTTTTAGACCAAGC<br>AAACCTGGACTCTCCATCGG | NM_001145750.2 | 149 |
| PGC-1α | ACCAGGACTCTGTATGGACTGA<br>TGAGGGCAATCCGTCTTCAT | NM_213963.2 | 299 |
| Nrf1 | GGCGGGAGGACCTTTTGTAT<br>ACTGTTGCCCCTGTACCAAC | XM_021079000.1 | 163 |
| SIRT3 | TGTGGTGTCGTTCATCTGTTG<br>CTTCCACAGACACCTGAGGC | NM_001110057.1 | 136 |
| TFAM | GCTCTCCGTTCAGTTTTGCG<br>GGAAGTTCCCTCCACAGCTC | NM_001130211.1 | 187 |
| OPA1 | ACAGAGGATGGTGCTTGTTGAC<br>CAGTATGATGGCGTTGGGATT | XM_047448216.1 | 130 |
| Mfn1 | AGAAAGCACAAAGCACAGGGGATG<br>CACTGCTGACTGCGAGATACACTC | XM_011512963.4 | 126 |
| Mfn2 | GCCACACCACCAACTGCTTCC<br>TCTTGACGCTCCTCTTCTCCTCTG | XM_047436156.1 | 96 |
| Drp1 | TCTGAATCTGGTGGGCATGATTGC<br>CTCCGCAGTAAAGGACTCGAAGTG | XM_001928848.6 | 91 |
| Fis1 | CTACCCAAAGGGAGCAAAGAG<br>GTCCAATGAGTCCAGCCAGTC | NM_016068.3 | 250 |
| Clpp | CGCTTATGACATCTACTCAC<br>GGGCTTCTTGTTGCTTTC | XM_003480818.4 | 130 |

| Gene Names | Sequence (5' → 3') | NCBI Reference Sequence | Amplicon size (bp) |
|---|---|---|---|
| HSP10 | GCAGCTGAAACGGTAACCAA<br>TCCACCCTTTCCTTTAGAGCC | NM_214307.1 | 111 |
| HSP60 | CGGATGCTGTAGCCGTTACT<br>ATCCCCAGCCTCTTCGTTTG | NM_001254716.1 | 191 |
| Htra-2 | GGCCGCGAAGTACCTATCTC<br>GCGTTGGTAACAATGAGCCC | XM_021087326.1 | 71 |
| ATF4 | AGTCCTTTTCTGCGAGTGGG<br>CTTCCACAGACACCTGAGGC | NM_001110057.1 | 80 |
| Parkin | GTGGTTCTGCGGTGGATTCTGAG<br>TTACTGCCTGTGGTTCTTTGGGAAG | NM_001044603.2 | 104 |
| PINK1 | CAGGAGCGGTCCCAAGCA<br>GTCATCACAGTGGCGAGGC | NM_032409.3 | 246 |
| LC3 | TGCCCTCAGACCGGCCTTTCAAGCA<br>TCCTTCTCCTGCTCATAGATGTCCG | XM_047440559.1 | 310 |
| FUNDC-1 | GGCAGCACCTGAAATCAACA<br>GCCTAGCAAAAAGCCTCCCA | XM_003135038.4 | 100 |
| BNIP3 | GACTCGCCAGGTTACAGACA<br>CAGTGACGTGCTTAGGACCC | XM_003359404.4 | 80 |
| Beclin1 | GGTTGGATCAGGAGGAAGCT<br>TGTCCACTGTGCCAAATGTG | NM_001044530.1 | 181 |
| Atg5 | AGAAACCTAGAGAGGGCCACA<br>TCTTCCTAGTCAAACAACGTCA | NM_001037152.2 | 111 |
| P62 | CGATGGCGATGTCGTATGTG<br>TTGCTGTGCTCCTTGTGAATG | XM_003123639.4 | 244 |

## Table S2. Total Variance Explained of PCA

**Total Variance Explained (Mitophagy)**

| Component | Initial Eigenvalues | | | Extraction Sums of Squared Loadings | | |
|---|---|---|---|---|---|---|
| | Total | % of Variance | Total | % of Variance | Total | % of Variance |
| 1 | 7.315 | 91.432 | 91.432 | 7.315 | 91.432 | 91.432 |
| 2 | .284 | 3.548 | 94.980 | .284 | 3.548 | 94.980 |
| 3 | .145 | 1.818 | 96.797 | | | |
| 4 | .096 | 1.201 | 97.998 | | | |
| 5 | .073 | .912 | 98.910 | | | |
| 6 | .055 | .684 | 99.594 | | | |
| 7 | .032 | .406 | 100.000 | | | |
| 8 | .-1.539E-16 | -1.924E-15 | 100.000 | | | |

Extraction Method: Principal Component Analysis (PCA).

**Figure S1. Original western blot gels used in the study**

| Claudin-3 (Fig.2F) | β-actin (Fig.2F) |
|---|---|
| 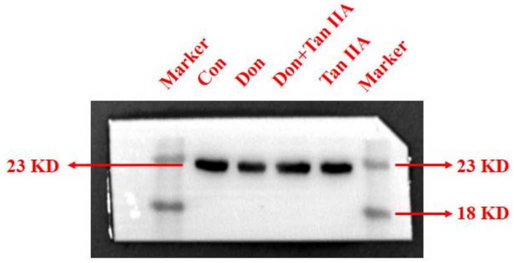 | 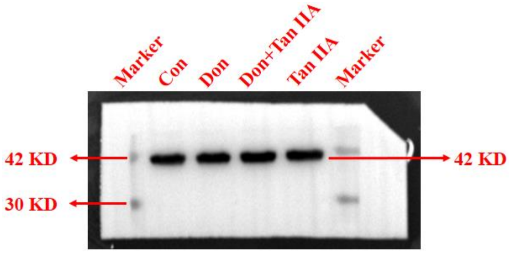 |
| **Claudin-1 (Fig.2G)** | **β-actin (Fig.2G)** |
| 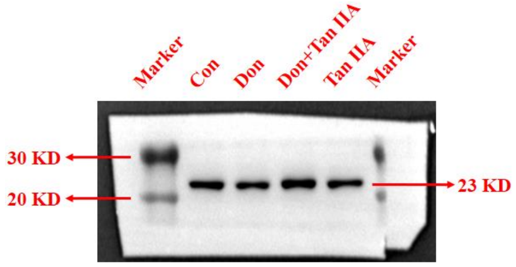 | 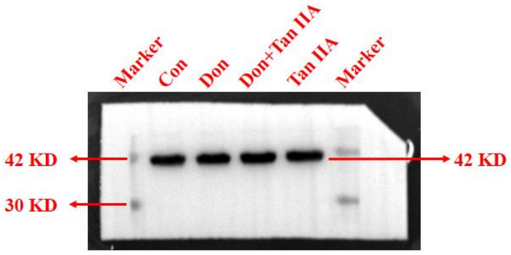 |
| **Occludin (Fig.2H)** | **β-actin (Fig.2H)** |
| 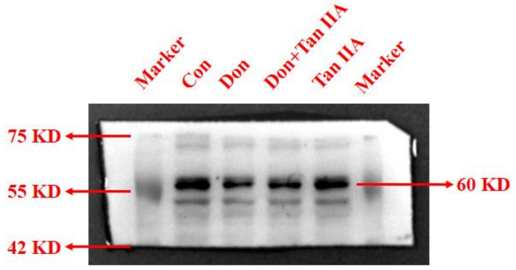 | 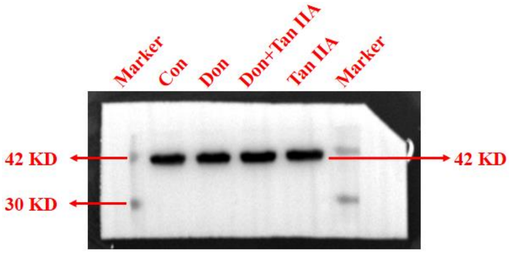 |
| **ZO-1 (Fig.2I)** | **β-actin (Fig.2I)** |
| 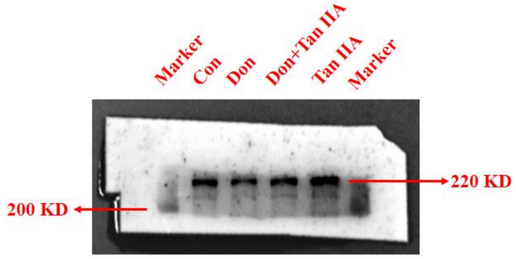 | 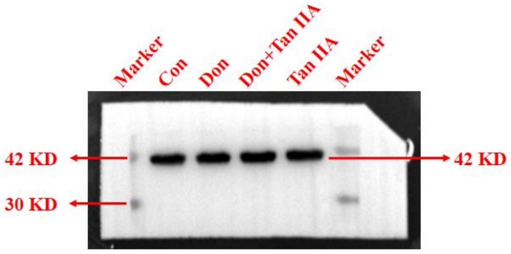 |
| **N-Cad (Fig.2J)** | **β-actin (Fig.2J)** |
| 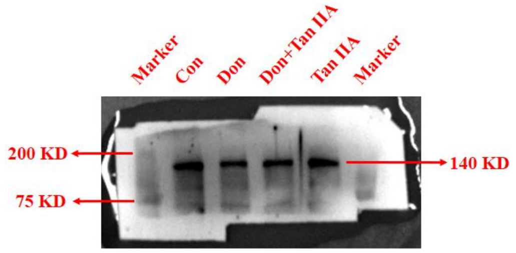 | 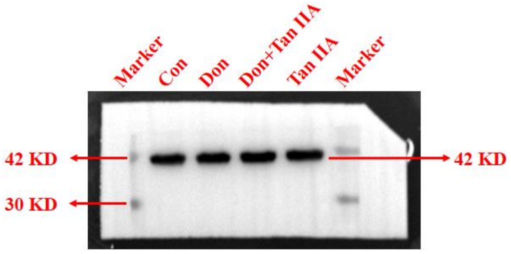 |

**LC3 I/II (Fig.6A)**

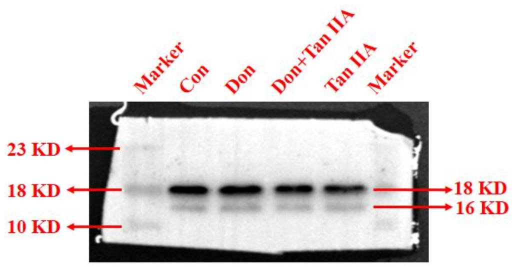


**β-actin (Fig.6A)**

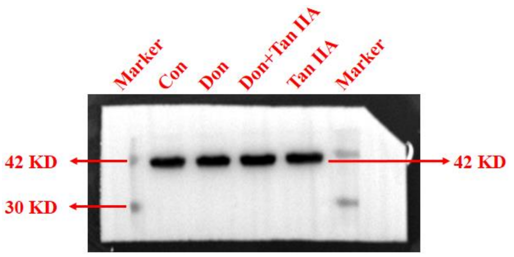


**Beclin1 (Fig.6B)**

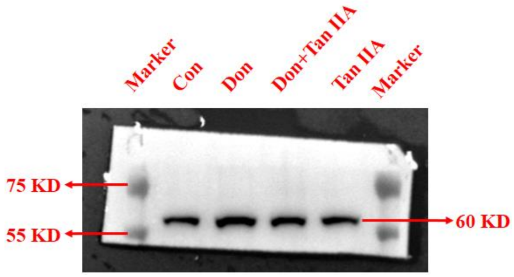


**β-actin (Fig.6B)**

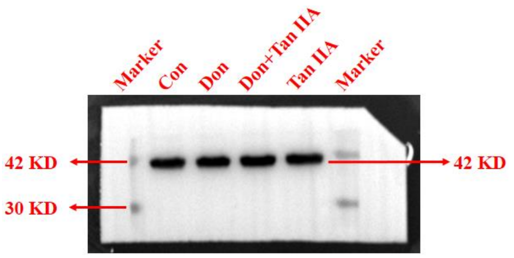


**P62 (Fig.6C)**

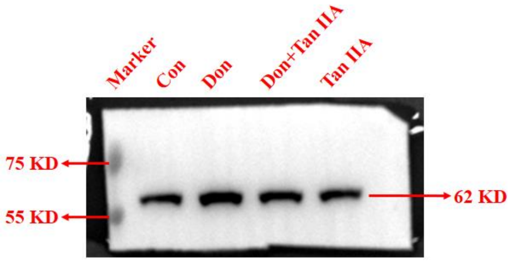


**β-actin (Fig.6C)**

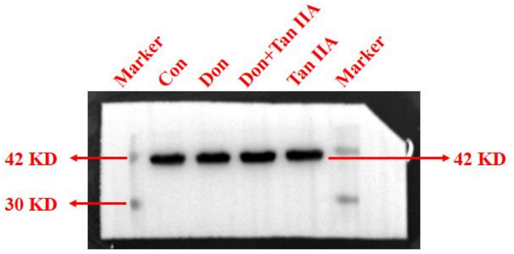


**Parkin (Fig.6D)**

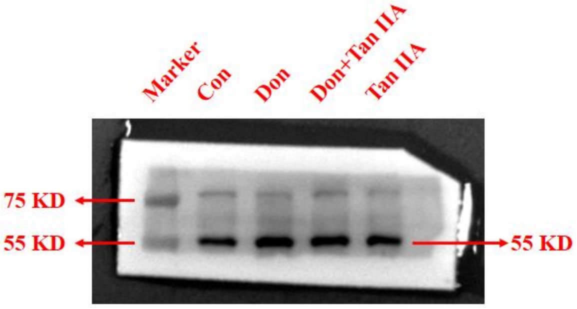


**β-actin (Fig.6D)**

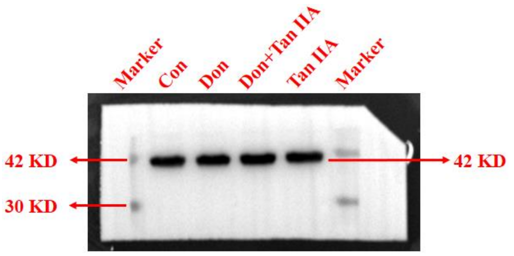


**PINK1 (Fig.6E)**

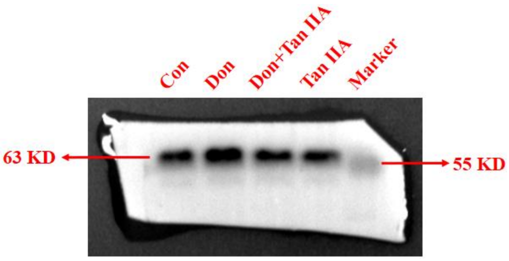


**β-actin (Fig.6E)**

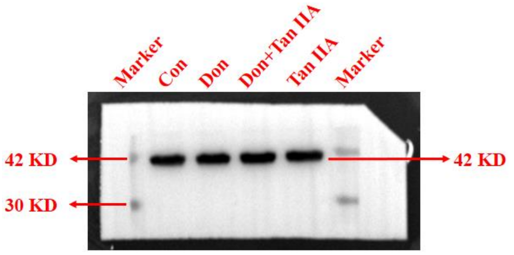

Supplement: Supplementary file 1 [file antioxidants-13-00121-s001.zip › antioxidants-2822802-supplementary.pdf]
